# Development and validation of a carotid atherosclerosis risk prediction model based on a Chinese population

**DOI:** 10.3389/fcvm.2022.946063

**Published:** 2022-08-02

**Authors:** Guoqing Huang, Qiankai Jin, Xiaoqing Tian, Yushan Mao

**Affiliations:** ^1^Department of Endocrinology, The Affiliated Hospital of Medical School, Ningbo University, Ningbo, China; ^2^School of Medicine, Ningbo University, Ningbo, China

**Keywords:** carotid atherosclerosis, independent risk factors, prediction model, early diagnosis, nomogram

## Abstract

**Purpose:**

This study aimed to identify independent risk factors for carotid atherosclerosis (CAS) and construct and validate a CAS risk prediction model based on the Chinese population.

**Methods:**

This retrospective study included 4,570 Chinese adults who underwent health checkups (including carotid ultrasound) at the Zhenhai Lianhua Hospital, Ningbo, China, in 2020. All the participants were randomly assigned to the training and validation sets at a ratio of 7:3. Independent risk factors associated with CAS were identified using multivariate logistic regression analysis. The least absolute shrinkage and selection operator combined with 10-fold cross-validation were screened for characteristic variables, and nomograms were plotted to demonstrate the risk prediction model. C-index and receiver operating characteristic curves, calibration plots, and decision curve analysis (DCA) were used to evaluate the risk model’s discrimination, calibration, and clinical applicability.

**Results:**

Age, body mass index, diastolic blood pressure, white blood cell count, mean platelet volume, alanine transaminase, aspartate transaminase, and gamma-glutamyl transferase were identified as independent risk factors for CAS. In the training, internal validation, and external validation sets, the risk model showed good discriminatory power with C-indices of 0.961 (0.953–0.969), 0.953 (0.939–0.967), and 0.930 (0.920–0.940), respectively, and excellent calibration. The results of DCA showed that the prediction model could be beneficial when the risk threshold probabilities were 1–100% in all sets. Finally, a network computer (dynamic nomogram) was developed to facilitate the physicians’ clinical operations. The website is https://nbuhgq.shinyapps.io/DynNomapp/.

**Conclusion:**

The development of risk models contributes to the early identification and prevention of CAS, which is important for preventing and reducing adverse cardiovascular and cerebrovascular events.

## Introduction

Atherosclerosis is a systemic atherosclerotic disease characterized by thickening, hardening, and loss of elasticity of the arterial walls and has become the pathological basis of many cardiovascular and cerebrovascular diseases, such as coronary heart disease and stroke (1). As part of systemic atherosclerosis, carotid atherosclerosis (CAS) has become an important window for observing systemic vascular health and the early risk of atherosclerosis due to its superficial location and ease of ultrasound manipulation (2, 3). In a global meta-analysis, approximately 28% of individuals in the general population (30–79 years) had carotid intimal thickening (4), potentially threatening people’s health.

As the global aging process increases, the incidence of stroke has become a leading cause of morbidity and mortality (5). CAS is a major and potentially preventable cause of ischemic stroke. Some studies have shown that CAS is associated with 20–30% of strokes (6). Diagnostic examination modalities for CAS include carotid ultrasound, computed tomography, magnetic resonance imaging, and invasive angiography. Carotid ultrasound is the primary screening modality for CAS (6). Additionally, studies have shown that serum biomarkers such as interleukin, homocysteine, and adipokines contribute to the early diagnosis of CAS (7). The basic clinical treatment options for CAS are lifestyle modification, control of cardiovascular risk factors, antiplatelet aggregation (requiring adequate assessment of bleeding risk), and lipid-lowering (8, 9), whereas surgical intervention is required for severe carotid intimal thickening.

Reducing the risk factors associated with CAS is crucial in reducing adverse cardiovascular events occurrence. A meta-analysis by Ji et al. (10) showed that hyperlipidemia, hyperhomocysteinemia, hypertension (HTN), hyperuricemia, smoking history, metabolic syndrome, hypertriglyceridemia, diabetes mellitus (DM), and low-density lipoprotein (LDL) were significantly associated with CAS. In addition, a global meta-analysis showed that gender (male), smoking history, DM, HTN, and dyslipidemia are strongly associated with CAS (4). Identifying risk factors is a guide to targeting the prevention and control of CAS.

Although numerous risk factors associated with CAS are already known, few clinical risk prediction models related to CAS have been reported in Chinese populations. The nomogram is a common visual presentation tool for disease risk prediction models that is user-friendly and easy to understand. The current study aimed to develop and validate an analytical predictive model for CAS based on a Chinese population using statistical algorithms. This study will play an important role in the early identification and prevention of CAS in the Chinese population.

## Materials and methods

### Patients

**FIGURE 1:**
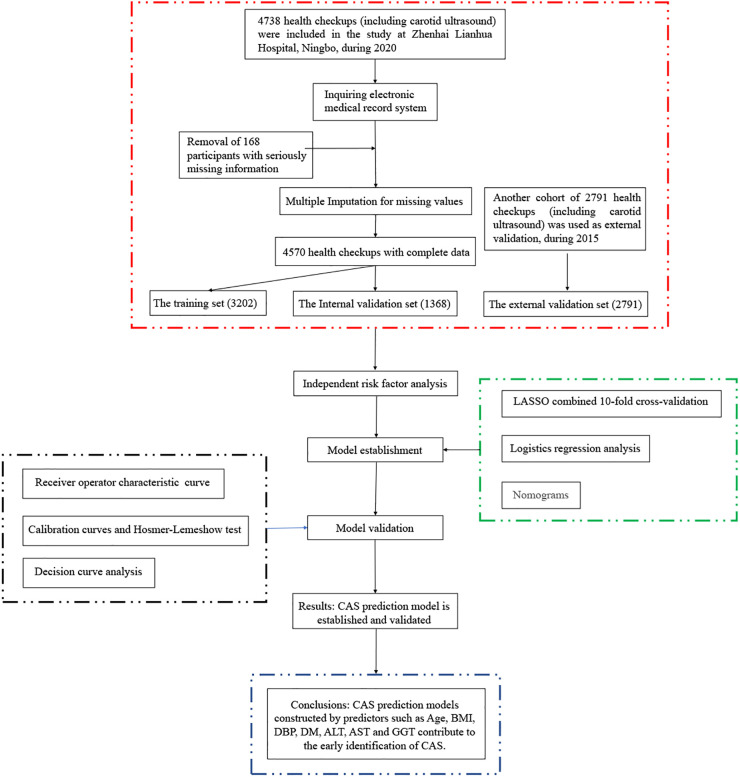
Flowchart of the participants.

A total of 4,738 adults (19–93 years) who underwent health checkups (including carotid ultrasound) at Zhenhai Lianhua Hospital, Ningbo, China, in 2020 were initially included in this study. Relevant information about the participants was obtained through the hospital’s electronic medical record system. Those with serious missing information (exceeding 20% of the total) were excluded, and those with less missing information (less than 20% of the total) were filled by multiple interpolations. Ultimately, 4,570 participants were included in this study. CAS was defined as an increase in carotid intima-media thickness of ≥1 mm or plaque formation (11). CAS diagnosis was based on carotid ultrasound results, recorded independently by 2 ultrasound physicians. The external validation dataset (2,791) was obtained from the 2015 health checkups (different from 2020). This study was approved by the ethics committee of the Affiliated Hospital of Medical School, Ningbo University, Ningbo, China (KY20191114). A flowchart of the participants is shown in Figure 1.

### Clinical baseline data

The methods and procedures for testing the clinical baseline data were based on previous studies (12–14). Gender, age, body mass index (BMI), systolic blood pressure (SBP), diastolic blood pressure (DBP), heart rate (HR), DM, HTN, Drinking and Smoking history, white blood cell count (WBC), neutrophil count, eosinophil count, basophil count, lymphocyte count, red blood cell count (RBC), hemoglobin, red blood cell distribution width, mean red blood cell volume, platelet count, platelet distribution width (PDW), mean platelet volume (MPV), alanine aminotransferase (ALT), aspartate aminotransferase (AST), total bilirubin, direct bilirubin, indirect bilirubin, total protein, albumin, globulin, gamma-glutamyl transpeptidase (GGT), total bile acids, blood urea nitrogen, serum creatinine, uric acid, fasting blood glucose, total cholesterol (TC), triglycerides, high-density lipoprotein, LDL, apolipoprotein A, apolipoprotein B (Apo-B), thyroid stimulating hormone, total triiodothyronine, total tetraiodothyronine, free triiodothyronine, and free tetraiodothyronine were obtained from the hospital electronic medical record system.

### Statistical analysis

This study enrolled 4,750 adults, and the measurement data were expressed as mean ± standard deviation; the count data were expressed as counts (%). Statistical analysis was performed with R software (version 4.1.2).^1^ All tests were two-tailed, and a *P*-value <0.05 was considered statistically significant.

Participants were randomly assigned to the training and internal validation sets at a certain ratio (7:3) (15). Independent risk factors were identified using multivariate logistic regression analysis. The least absolute shrinkage and selection operator (LASSO) combined with 10-fold cross-validation was used to screen for characteristic variables associated with CAS. The visual presentation of the risk prediction model was displayed using a nomogram. Risk prediction models were evaluated in terms of discrimination (C-index and receiver operating characteristic (ROC) curves), calibration ability (Hosmer–Lemeshow test and calibration curves), and clinical applicability [decision curve analysis (DCA)] in the training, internal validation, and external validation sets, respectively.

## Results

### Characteristics of the study population

This study enrolled 4,570 participants (3,010 males and 1,560 females). The detection rate of CAS by carotid ultrasound in 2020 was 83.4% (3,813) compared with 73.1% (2,039) in 2015 (external validation set) at Zhenhai Lianhua Hospital, Ningbo, China. The total study population was divided into training (3,202) and internal validation (1,358) sets according to a 7:3 ratio, while an external validation set (2,791) was introduced to ensure the stability of the model (Table 1). Univariate analysis showed a higher proportion of males in the CAS group than in the healthy control (HC) group (66.90% vs. 60.63%, P < 0.001). The mean age of the HC and CAS groups was 40.08 and 64.37 years, respectively. The CAS group had higher proportions of DM, HTN, smoking history, and drinking history than the HC group. In addition, there were differences between the 2 groups in terms of routine blood tests, liver function, lipids, glucose, and thyroid function. The baseline information of the study cohort is shown in Table 2.

**TABLE 1:** Characteristics of participants in different cohorts.

	Training set	Internal validation set	External validation set
N	3202	1368	2791
Sex (male)	2116 (66.08)	894 (65.35)	1903 (68.18)
Age, years	60.30 (14.33)	60.46 (14.21)	59.90 (13.36)
BMI, kg/m^2^	23.57 (3.05)	23.52 (2.97)	23.12 (2.83)
SBP, mmHg	136.06 (18.28)	136.62 (18.84)	131.40 (18.61)
DBP, mmHg	79.37 (11.16)	79.64 (11.56)	76.76 (11.32)
HR, times/min	78.53 (12.60)	78.87 (12.51)	78.88 (12.30)
DM	34 (1.06)	18 (1.32)	124 (4.44)
HTN	99 (3.09)	39 (2.95)	450 (16.12)
Drinking history	192 (6.00)	94 (6.87)	849 (30.42)
Smoking history	175 (5.47)	83 (6.07)	679 (24.33)
WBC, 10^9/L	5.93 (1.52)	5.97 (1.51)	5.98 (1.52)
NEC, 10^9/L	3.43 (1.14)	3.48 (1.15)	3.42 (1.14)
EOC, 10^9/L	0.15 (0.15)	0.14 (0.13)	0.15 (0.14)
BAC, 10^9/L	0.02 (0.01)	0.02 (0.01)	0.01 (0.01)
LYC, 10^9/L	1.96 (0.61)	1.96 (0.60)	2.05 (0.61)
RBC, 10^12/L	4.79 (0.50)	4.82 (0.50)	4.61 (0.45)
HGB, g/L	145.59 (14.83)	146.10 (15.04)	140.14 (14.16)
RDW, %	12.75 (0.92)	12.78 (1.00)	12.83 (0.87)
MCV, fl	92.89 (5.30)	92.55 (5.65)	92.94 (4.98)
PLT, 10^9/L	221.85 (58.54)	221.56 (55.23)	205.81 (52.34)
PDW, %	13.58 (2.33)	13.53 (2.30)	12.43 (1.99)
MPV, fl	11.00 (0.99)	10.97 (0.98)	10.40 (0.93)
ALT, U/L	24.18 (17.38)	24.52 (17.93)	19.01 (12.69)
AST, U/L	25.79 (12.66)	26.42 (14.49)	22.77 (10.01)
T-BIL, μmol/L	14.71 (6.12)	14.58 (6.06)	14.77 (6.34)
D-BIL, μmol/L	3.55 (1.77)	3.54 (1.81)	4.56 (1.60)
I-BIL, μmol/L	11.16 (4.84)	11.04 (4.73)	10.20 (4.94)
TP, g/L	74.21 (4.03)	74.24 (4.08)	72.52 (4.32)
ALB, g/L	44.79 (2.22)	44.76 (2.22)	44.99 (2.62)
GLOB, g/L	29.42 (3.74)	29.48 (3.56)	27.53 (4.18)
GGT, U/L	35.99 (46.92)	36.55 (44.48)	31.12 (35.03)
TBA, μmol/L	4.01 (3.93)	4.16 (5.17)	2.70 (2.90)
BUN, mmol/L	5.36 (1.54)	5.31 (1.55)	5.17 (1.52)
Scr, μmol/L	73.26 (36.99)	72.45 (36.39)	61.58 (18.63)
UA, μmol/L	356.77 (86.33)	357.98 (86.30)	326.81 (80.18)
FBG, mmol/L	5.90 (1.38)	5.90 (1.40)	5.37 (1.15)
TC, mmol/L	5.22 (1.12)	5.23 (1.09)	4.86 (0.98)
TG, mmol/L	1.57 (1.10)	1.56 (1.13)	1.35 (0.91)
HDL, mmol/L	1.28 (0.39)	1.31 (0.39)	1.52 (0.28)
LDL, mmol/L	2.99 (0.89)	2.97 (0.87)	2.70 (0.73)
Apo-A, g/L	1.45 (0.27)	1.47 (0.27)	1.54 (0.34)
Apo-B, g/L	0.99 (0.28)	0.98 (0.28)	0.70 (0.18)
TSH, mIU/L	2.11 (2.09)	2.14 (2.25)	2.09 (1.84)
TT3, nmol/L	1.59 (0.32)	1.60 (0.34)	1.64 (0.25)
TT4, nmol/L	116.70 (20.01)	117.75 (22.91)	113.37 (19.08)
FT3, pmol/L	5.15 (0.64)	5.17 (0.66)	4.56 (0.49)
FT4, pmol/L	11.19 (1.68)	11.24 (1.68)	11.10 (1.54)

BMI, body mass index; SBP, systolic blood pressure; DBP, diastolic blood pressure; HR, heart rate; DM, diabetes mellitus; HTN, hypertension; WBC, white blood cell count; NET, neutrophil count; EOC, eosinophil count; BAC, basophil count; LYC, lymphocyte count; RBC, red blood cell count; HGB, hemoglobin; RDW, red blood cell distribution width; MCV, mean red blood cell volume; PLT, platelet count; PDW, platelet distribution width; MPV, mean platelet volume; ALT, alanine aminotransferase; AST, aspartate aminotransferase; T-BIL, total bilirubin; D-BIL, direct bilirubin; I-BIL, indirect bilirubin; TP, total protein; ALB, albumin; GLOB, globulin; GGT, gamma-glutamyl transpeptidase; TBA, total bile acids; BUN, blood urea nitrogen; Scr, serum creatinine; UA, uric acid; FBG, fasting blood glucose; TC, total cholesterol; TG, triglycerides; HDL, high-density lipoprotein; LDL, low-density lipoprotein; Apo-A, apolipoprotein -A; Apo-B, apolipoprotein-B; TSH, thyroid stimulating hormone; TT3, total triiodothyronine; TT4, total tetraiodothyronine; FT3, free triiodothyronine; FT4, free tetraiodothyronine.

**TABLE 2:** Univariate analysis of carotid atherosclerosis.

	Overall	HC	CAS	P-value
N	4570	757	3813	
Sex (male)	3010 (65.86)	459 (60.63)	2551 (66.90)	0.001
Age, years	60.35 (14.29)	40.08 (10.91)	64.37 (11.11)	<0.001
BMI, kg/m^2^	23.55 (3.03)	21.84 (2.54)	23.89 (3.00)	<0.001
SBP, mmHg	136.22 (18.45)	120.52 (13.91)	139.34 (17.63)	<0.001
DBP, mmHg	79.45 (11.28)	73.82 (9.99)	80.57 (11.19)	<0.001
HR, times/min	78.63 (12.57)	79.50 (12.48)	78.46 (12.58)	0.036
DM	52 (1.13)	0 (0.0)	52 (1.36)	<0.001
HTN	138 (3.01)	3 (0.40)	135 (3.54)	<0.001
Drinking history	286 (6.26)	83 (10.96)	203 (5.32)	<0.001
Smoking history	258 (5.64)	69 (9.11)	189 (4.96)	<0.001
WBC, 10^9/L	5.94 (1.52)	5.69 (1.40)	5.99 (1.54)	<0.001
NEC, 10^9/L	3.45 (1.15)	3.24 (1.07)	3.49 (1.16)	<0.001
EOC, 10^9/L	0.15 (0.14)	0.14 (0.11)	0.15 (0.14)	0.023
BAC, 10^9/L	0.02 (0.01)	0.02 (0.01)	0.02 (0.01)	0.545
LYC, 10^9/L	1.96 (0.61)	1.95 (0.55)	1.96 (0.62)	0.536
RBC, 10^12/L	4.80 (0.50)	4.88 (0.49)	4.79 (0.50)	<0.001
HGB, g/L	145.75 (14.90)	145.41 (15.45)	145.81 (14.78)	0.496
RDW, %	12.76 (0.95)	12.65 (1.09)	12.78 (0.91)	<0.001
MCV, fl	92.79 (5.41)	91.08 (5.49)	93.13 (5.33)	<0.001
PLT, 10^9/L	221.76 (57.56)	235.50 (54.53)	219.03 (57.76)	<0.001
PDW, %	13.56 (2.32)	13.80 (2.46)	13.51 (2.29)	0.002
MPV, fl	10.99 (0.99)	11.11 (1.02)	10.97 (0.98)	<0.001
ALT, U/L	24.28 (17.55)	19.60 (13.17)	25.21 (18.15)	<0.001
AST, U/L	25.98 (13.24)	22.08 (7.91)	26.75 (13.93)	<0.001
T-BIL, μmol/L	14.67 (6.11)	14.36 (6.16)	14.73 (6.09)	0.122
D-BIL, μmol/L	3.54 (1.78)	3.51 (1.74)	3.55 (1.79)	0.581
I-BIL, μmol/L	11.13 (4.80)	10.85 (4.77)	11.18 (4.81)	0.078
TP, g/L	74.22 (4.04)	74.16 (3.68)	74.23 (4.11)	0.68
ALB, g/L	44.78 (2.22)	45.76 (2.10)	44.59 (2.19)	<0.001
GLOB, g/L	29.44 (3.69)	28.41 (3.20)	29.64 (3.74)	<0.001
GGT, U/L	36.16 (46.20)	24.43 (22.53)	38.48 (49.24)	<0.001
TBA, μmol/L	4.06 (4.34)	3.34 (2.82)	4.20 (4.57)	<0.001
BUN, mmol/L	5.34 (1.54)	4.87 (1.16)	5.44 (1.59)	<0.001
Scr, μmol/L	73.01 (36.81)	68.14 (12.39)	73.98 (39.85)	<0.001
UA, μmol/L	357.13 (86.31)	332.13 (82.22)	362.10 (86.25)	<0.001
FBG, mmol/L	5.90 (1.39)	5.21 (0.76)	6.04 (1.44)	<0.001
TC, mmol/L	5.22 (1.11)	5.01 (0.98)	5.26 (1.13)	<0.001
TG, mmol/L	1.56 (1.11)	1.23 (1.03)	1.63 (1.11)	<0.001
HDL, mmol/L	1.29 (0.39)	1.38 (0.38)	1.28 (0.39)	<0.001
LDL, mmol/L	2.98 (0.89)	2.83 (0.77)	3.01 (0.91)	<0.001
Apo-A, g/L	1.46 (0.27)	1.46 (0.25)	1.46 (0.27)	0.794
Apo-B, g/L	0.98 (0.28)	0.89 (0.24)	1.00 (0.28)	<0.001
TSH, mIU/L	2.12 (2.14)	1.88 (1.03)	2.16 (2.30)	0.001
TT3, nmol/L	1.59 (0.32)	1.63 (0.34)	1.58 (0.32)	<0.001
TT4, nmol/L	117.01 (20.92)	111.20 (18.72)	118.17 (21.15)	<0.001
FT3, pmol/L	5.16 (0.65)	5.35 (0.69)	5.12 (0.63)	<0.001
FT4, pmol/L	11.21 (1.68)	11.28 (1.51)	11.19 (1.71)	0.218

BMI, body mass index; SBP, systolic blood pressure; DBP, diastolic blood pressure; HR, heart rate; DM, diabetes mellitus; HTN, hypertension; WBC, white blood cell count; NET, neutrophil count; EOC, eosinophil count; BAC, basophil count; LYC, lymphocyte count; RBC, red blood cell count; HGB, hemoglobin; RDW, red blood cell distribution width; MCV, mean red blood cell volume; PLT, platelet count; PDW, platelet distribution width; MPV, mean platelet volume; ALT, alanine aminotransferase; AST, aspartate aminotransferase; T-BIL, total bilirubin; D-BIL, direct bilirubin; I-BIL, indirect bilirubin; TP, total protein; ALB, albumin; GLOB, globulin; GGT, gamma-glutamyl transpeptidase; TBA, total bile acids; BUN, blood urea nitrogen; Scr, serum creatinine; UA, uric acid; FBG, fasting blood glucose; TC, total cholesterol; TG, triglycerides; HDL, high-density lipoprotein; LDL, low-density lipoprotein; Apo-A, apolipoprotein -A; Apo-B, apolipoprotein-B; TSH, thyroid stimulating hormone; TT3, total triiodothyronine; TT4, total tetraiodothyronine; FT3, free triiodothyronine; FT4, free tetraiodothyronine.

### Independent risk factors

Based on univariate analysis (Table 1), we selected candidate variables with P < 0.1 to screen independent risk factors. Covariance analysis among candidate variables showed variance inflation factors (VIF) > 10 for PDW, MPV, TC, LDL, and Apo-B. When the Akaike information criterion was −12180.32, the stepwise backward logistic regression analysis found the optimal model to include 20 variables such as age, BMI, HR, SBP, and DBP (VIF < 10). Furthermore, we constructed logistic regression equations.


(1) $$\log\left[\frac{P(\text{Carotid\_at}\hat{\text{herosc}}\text{lerosis}\;=\;1)}{1-P(\text{Carotid\_at}\hat{\text{heros}}\text{clerosis}\;=\;1)}\right]$$

$$\begin{array}{cc} & -19.05+0.22\,\left(\text{Age}\right)+0.2\,\left(\text{BMI}\right)-\\ & 0.01\,\left(\text{HR}\right)+0.01\,\left(\text{SBP}\right)+0.03\,\left(\text{DBP}\right)-\\ & 0.41\,\left({\text{Drinking\_history}}_{\text{Yes}}\right)+\\ & 0.31\,\left({\text{Smoking\_history}}_{\text{Yes}}\right)+0.11\,\left(\text{WBC}\right)-\\ & 0.01\,\left(\text{RBC}\right)+0.01\,\left(\text{MRBCV}\right)-0.17\,\left(\text{MPV}\right)+\\ & 0.04\,\left(\text{ALT}\right)-0.04\,\left(\text{AST}\right)+0\,\left(\text{I\_BIL}\right)+\\ & 0.02\,\left(\text{GLOB}\right)+0.01\,\left(\text{GGT}\right)+0.11\,\left(\text{FBG}\right)-\\ & 0.2\,\left(\text{LDL}\right)+1.53\,\left(\text{Apo\_B}\right)+0.2\,\left(\mathrm{TT3}\right) \end{array}$$

Where BMI, body mass index; HR, heart rate; SBP, systolic blood pressure; DBP, diastolic blood pressure; WBC, white blood cell count; RBC, red blood cell count; MRBCV, mean red blood cell velocities; MPV, mean platelet volume; ALT, alanine transaminase; AST, aspartate transaminase; I_BIL, indirect bilirubin; GLOB, globulin; GGT, gamma-glutamyl transpeptidase; FBG, fasting blood glucose; LDL, low-density lipoprotein; Apo_B, apolipoprotein B; TT3, total triiodothyronine.

Independent risk factors associated with CAS were also identified, including age, BMI, WBC count, MPV, ALT, AST, and GGT (Table 3).

**TABLE 3:** Multivariate logistic regression analysis.

Variable	Coefficients	Odds ratio (95% CI)	P-value
Age	0.222	1.249 (1.225–1.274)	<0.001
BMI	0.200	1.221 (1.156–1.291)	<0.001
HR	–0.007	0.993 (0.982–1.004)	0.22786
SBP	0.007	1.007 (0.994–1.021)	0.31200
DBP	0.027	1.029 (1.008–1.048)	<0.01
Drinking history	–0.412	0.662 (0.393–1.117)	0.12159
Smoking history	0.305	1.357 (0.782–2.390)	0.28334
WBC	0.114	1.121 (1.018–1.236)	<0.05
RBC	–0.009	0.991 (0.684–1.435)	0.96104
MCV	0.006	1.006 (0.978–1.035)	0.67506
MPV	–0.170	0.844 (0.738–0.966)	<0.05
ALT	0.036	1.036 (1.020–1.053)	<0.001
AST	–0.037	0.964 (0.947–0.984)	<0.001
I-BIL	–0.002	0.998 (0.970–1.028)	0.908005
GLOB	0.024	1.024 (0.982–1.069)	0.26725
GGT	0.007	1.007 (1.003–1.013)	<0.05
FBG	0.098	1.112 (0.979–1.289)	0.12646
LDL	–0.200	0.765 (0.503–1.325)	0.41725
Apo-B	1.531	4.623 (0.922–23.683)	0.06452
TT3	0.198	1.219 (0.782–1.915)	0.38678

BMI, body mass index; HR, heart rate; SBP, systolic blood pressure; DBP, diastolic blood pressure; WBC, white blood cell count; RBC, red blood cell count; MCV, mean red blood cell volume; MPV, mean platelet volume; ALT, alanine aminotransferase; AST, aspartate aminotransferase; I-BIL, indirect bilirubin; GLOB, globulin; GGT, gamma-glutamyl transpeptidase; FBG, fasting blood glucose; LDL, low-density lipoprotein; Apo-B, apolipoprotein-B; TT3, total triiodothyronine.

### Construction of predictive models

**FIGURE 2:**
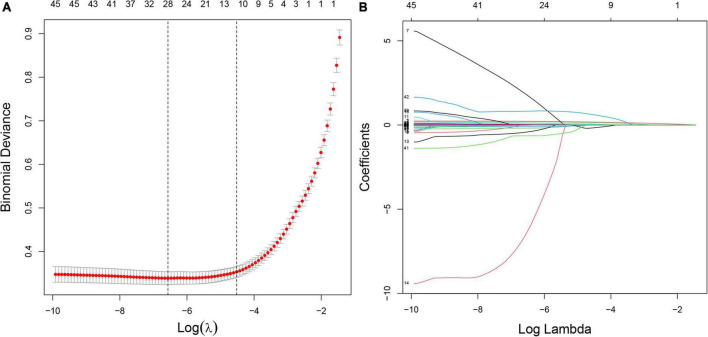
Screening of characteristic variables using the LASSO regression analysis. **(A)** The selection of the best parameter (lambda) in the LASSO model uses 10-fold cross-validation with the lowest standard. The relationship curve between partial likelihood deviation (binomial deviation) and log (lambda) was plotted. Dotted vertical lines were drawn at the optimal values by using the minimum criteria and the 1 SE of the minimum criteria (the 1 SE criteria). **(B)** LASSO coefficient profiles of the 28 characteristic variables. A coefficient profile plot was produced against the log (lambda) sequence. LASSO, least absolute shrinkage and selection operator; SE, standard error.

**FIGURE 3:**
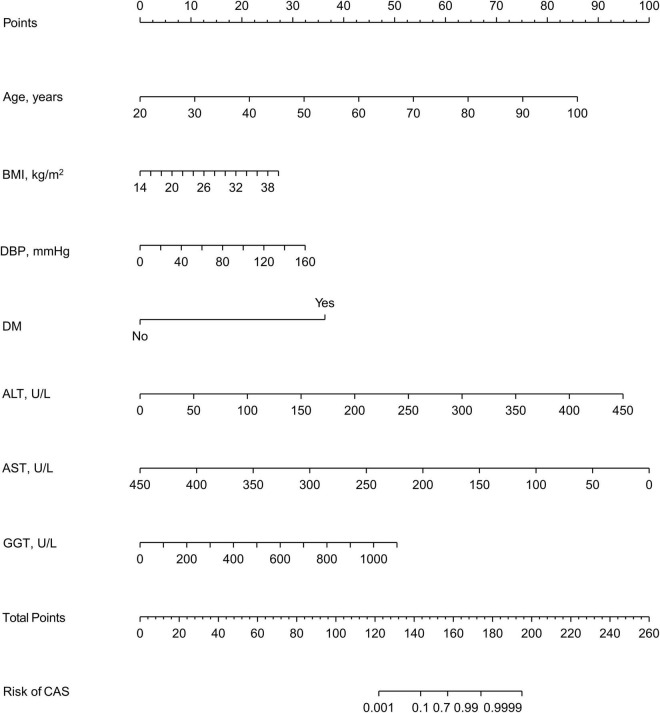
A nomogram for predicting the probability of the development of CAS. The nomogram is used by scoring each variable on its corresponding score scale. The scores for all variables are then summed up to obtain the total score, and a vertical line is drawn from the total point row to indicate the estimated probability of the development of CAS. carotid atherosclerosis, CAS; body mass index, BMI; systolic blood pressure, SBP; diastolic blood pressure, DBP; diabetes mellitus, DM; alanine aminotransferase, ALT; aspartate aminotransferase, AST; gamma-glutamyl transpeptidase, GGT.

**FIGURE 4:**
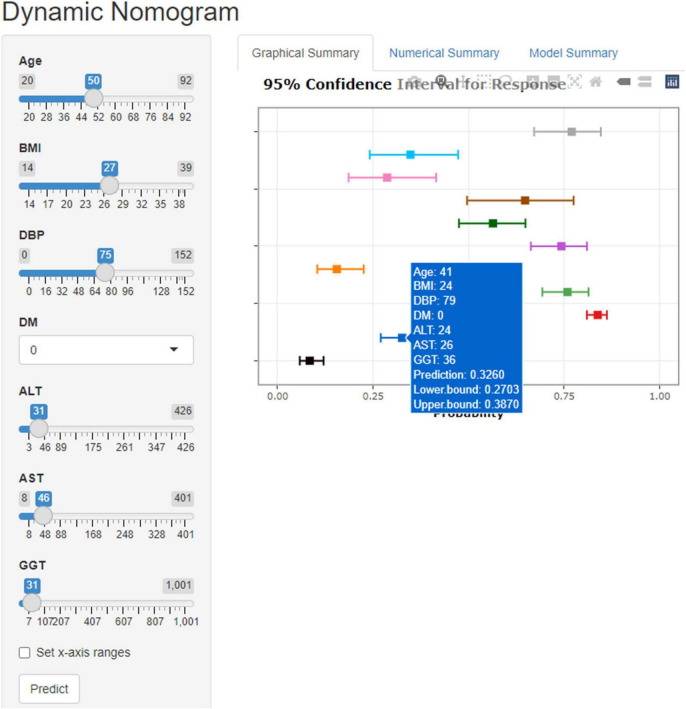
The web version of the dynamic nomogram.

In the training set, 28 non-zero characteristic variables were screened using LASSO regression analysis (Figure 2 and Table 4). Low-weight variables (points < 20) were removed from the risk prediction model. Finally, we selected age, BMI, DBP, DM, ALT, AST, and GGT for model construction (Figure 3). In addition, we developed a web version of the dynamic nomogram (Figure 4) for ease of daily use. The URL is https://nbuhgq.shinyapps.io/DynNomapp/.

**TABLE 4:** Coefficients and lambda.min value of the LASSO regression.

Variables	Coefficients	Lambda.min
Age	0.2196	0.0014
BMI	0.1777	
SBP	0.0097	
DBP	0.0293	
HR	–0.0040	
DM	1.8408	
Drinking history	–0.1880	
WBC	0.1084	
NEC	0.0220	
EOC	–0.4282	
BAC	–6.5259	
PLT	0.0014	
MPV	–0.0999	
ALT	0.0241	
AST	–0.0176	
I-BIL	0.0032	
ALB	–0.0096	
GLOB	0.0248	
GGT	0.0051	
Scr	0.0013	
UA	0.0020	
FBG	0.0118	
TG	0.0242	
Apo-A	–0.6405	
Apo-B	0.8163	
TT3	0.0375	
TT4	0.0082	
FT4	–0.0283	

BMI, body mass index; HR, heart rate; SBP, systolic blood pressure; DBP, diastolic blood pressure; DM, diabetes mellitus; WBC, white blood cell count; NET, neutrophil count; EOC, eosinophil count; BAC, basophil count; PLT, platelet count; MPV, mean platelet volume; ALT, alanine aminotransferase; AST, aspartate aminotransferase; I-BIL, indirect bilirubin; ALB, albumin; GLOB, globulin; GGT, gamma-glutamyl transpeptidase; Scr, serum creatinine; FBG, fasting blood glucose; TG, triglycerides; LDL, low-density lipoprotein; Apo-A, apolipoprotein -A; Apo-B, apolipoprotein-B; TT3, total triiodothyronine; TT4, total tetraiodothyronine; FT4, free tetraiodothyronine.

### Validation of predictive models

**FIGURE 5:**
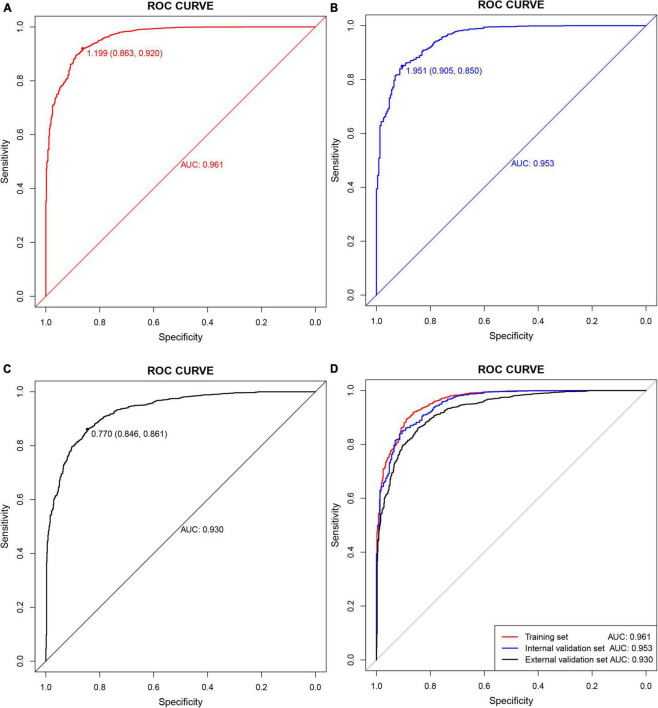
Receiver operating characteristic (ROC) curves. **(A)** Training set; **(B)** internal validation set; **(C)** external validation set; **(D)** all sets.

The C-index and area under the ROC curve (AUC) were used to assess the discriminatory ability of the risk model. The C-index was 0.961 (0.953–0.969), 0.953 (0.939–0.967), and 0.930 (0.920–0.940) in the training, internal validation, and external validation sets, respectively (Table 5), whereas the AUC was 0.961, 0.953, and 0.930, respectively (Figure 5).

**TABLE 5:** C-index in the study cohort.

	C-index (95%CI)	Dxy	aDxy	SD	Z	*P*-value
Training set	0.961 (0.953–0.969)	0.921	0.921	0.008	112.93	0
Internal validation set	0.953 (0.939–0.967)	0.905	0.905	0.028	23.15	0
External validation set	0.930 (0.920–0.940)	0.860	0.860	0.010	84.51	0

**FIGURE 6:**
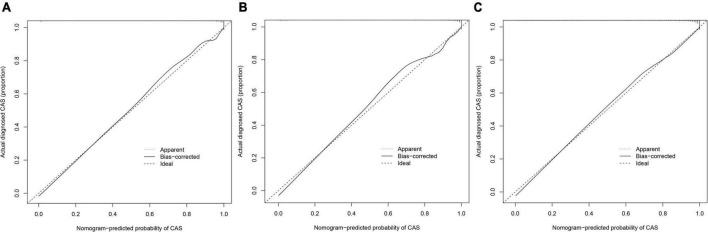
Calibration curves. The *x*-axis represents the predicted CAS risk. The *y*-axis represents the actual diagnosed CAS. The diagonal dotted line represents a perfect prediction by an ideal model. The solid line represents the performance of the nomogram, of which a closer fit to the diagonal dotted line represents a better prediction. **(A)** Training set; **(B)** internal validation set; **(C)** external validation set.

From the calibration curves, we observed that the predicted values were very close to the theoretical values in the training, internal validation, and external validation sets, showing a very good fit (Figure 6), which was further confirmed by the Hosmer-Lemeshow test (*P* > 0.05) (Table 6).

**TABLE 6:** Hosmer–Lemeshow test.

	Training set	Internal validation set	External validation set
χ^2^	12.9146	13.1931	16.7528
*P*-value	0.1665	0.1541	0.0527

**FIGURE 7:**
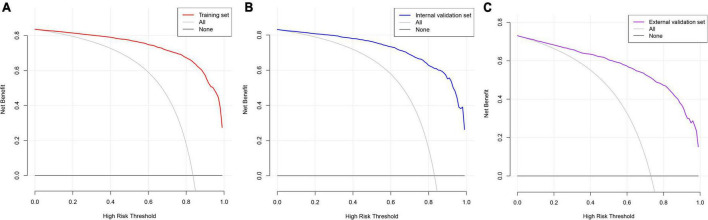
Decision curve analysis. The black straight line represents the net benefit when none of the participants are considered to develop CAS, while the light gray curve represents the net benefit when all participants are considered to develop CAS. The area between the color (red, blue, and purple) curve and the light gray curve in the model curve indicates the clinical utility of the model. **(A)** Training set; **(B)** internal validation set; **(C)** external validation set.

Decision curve analysis is often used to assess the clinical applicability of risk-prediction models. Figure 7 shows that the risk threshold probabilities for the training, internal validation, and external validation sets were 1–100%, suggesting that the risk prediction model is beneficial in this range.

## Discussion

With the popularity of ultrasound in health checkups, CAS detection rate is gradually increasing. The carotid ultrasound CAS detection rate in 2020 was 83.4% (3,813) compared to 73.1% (2,039) in 2015 (external validation set) at Zhenhai Lianhua Hospital in Ningbo, China. Age (odds ratio [OR]: 1.249 [1.225–1.274], *P* < 0.001), BMI (OR: 1.221 [1.156–1.291], *P* < 0.001), DBP (OR: 1.029 [1.008–1.048], *P* < 0.01), WBC (OR: 1.121 [1.018–1.236], *P* < 0.05), MPV (OR: 0.844 [0.738–0.966], *P* < 0.05), ALT (OR: 1.036 [1.020–1.053], *P* < 0.001), AST (OR: 0.964 [0.947–0.984], *P* < 0.001), GGT (OR: 1.007 [1.003–1.013], *P* < 0.05) were identified as independent risk factors for CAS. After removing the low-weight variables, age, BMI, DBP, DM, ALT, AST, and GGT were filtered out to design risk prediction models. The prediction model showed excellent clinical differentiation with C-indexes of 0.961 (0.953–0.969), 0.953 (0.939–0.967), and 0.930 (0.920–0.940) and degrees of fit in the training, internal validation, and external validation sets, respectively. In addition, DCA showed that the prediction model could benefit people with a risk threshold of 1%–100%.

Our study showed that age and DBP were risk factors for CAS, consistent with previous studies (4, 16). Previous studies have shown that BMI and abnormal lipid metabolism are closely related (17, 18) and are an important cause of CAS. This study found BMI to be an independent risk factor for CAS, consistent with a national cross-sectional study in China (11). However, a study in Iceland showed a negative association between BMI and CAS, which may be related to geographic location, ethnicity, diet, and lifestyle (19). Consistent with previous studies (20), we also found WBC count to be an independent risk factor for CAS in our study. CAS is gradually gaining acceptance as an inflammation-associated disease, and studies have shown that inflammatory markers such as interleukin 6 and C-reactive protein are serum markers of CAS (21). In addition, a randomized controlled trial has shown that anti-inflammatory therapy helps reduce the incidence of major cardiovascular adverse events (22). As the pathological basis of atherosclerosis, platelets promote the development and progression of atherosclerosis (23), which was also observed in our study. AST, ALT, and GGT levels are commonly used as indicators of liver dysfunction in clinical practice. In recent years, there has been an increasing number of studies on liver enzymes and cardiovascular risk, and a prospective meta-analysis revealed ALT and GGT levels in relation to cardiovascular disease risk (24). A study by Abdou et al. (25) based on a small sample of abdominally obese people (50) showed that AST was negatively associated with CAS; however, in our study, it was the opposite. A study in rural northeastern China showed that higher levels of education and income were associated with a lower risk of CAS (*P* < 0.05), although this relationship was absent after correction for confounders (26). The role of exercise in CAS remains controversial. A 6-year clinical trial showed that aerobic physical activity did not slow the progression of CAS (27) and might contribute to carotid endothelial injury (28). However, some studies have shown that lack of exercise is a risk factor for CAS (11, 26), and aerobic exercise could help combat carotid intima-media thickening in obese patients (29). In addition, a study in China showed that geographic location (rural areas) is associated with CAS (11).

Early identification and prevention of CAS are essential in reducing adverse cerebrovascular disease occurrence. The diagnosis of CAS in clinical practice relies mainly on carotid ultrasonography. However, there are still difficulties in the large-scale availability of ultrasound in health checkups owing to limited medical resources. The development of risk prediction models provides an alternative method for CAS detection that could benefit populations in less medically developed regions or countries. In addition, risk models could help physicians selectively perform further tests, which is beneficial in terms of saving health care resources. Xing et al. (30) constructed a risk prediction model for atherosclerosis in a systemic lupus erythematosus population based on RNA sequencing, and the model exhibited excellent clinical predictive value (AUC:0.922). However, this model has some limitations. First, the study’s sample size was limited (67); furthermore, it was not validated with internal and external samples, and the cost of RNA sequencing in atherosclerosis diagnosis was too high. In addition, an atherosclerosis prediction was constructed based on operational research (31), but it is too obscure and clinically inoperative.

Data mining in big clinical data provides technical support for establishing risk-prediction models (32). Nomograms can generate individual probabilities of clinical events by integrating different outcome and predictor variables and merging biological information with clinical prediction models (33–35). In recent years, it has been widely used as a prediction method in clinical settings (36, 37), and it plays a role in promoting personalized medicine (38, 39) and facilitates physicians in predicting disease risk (40). There have been many studies on CAS prevalence and risk factors (4, 41), but few studies on CAS risk-prediction models have been reported. LASSO is a widely used algorithm in machine learning to filter characteristics and interpretable predictors from a large number of potentially co-linear variables by constructing a penalty function (42, 43). LASSO combined with 10-fold cross-validation was used to screen for the characteristic variables associated with CAS. After several modeling attempts, we removed weakly weighted variables in the model (points < 20). Ultimately, 7 clinically common indicators, including age, BMI, DBP, DM, ALT, AST, and GGT, were screened as predictors to construct a risk prediction model. A network computer (dynamic nomogram) was developed to increase the tractability of the model. Our risk prediction model showed a high predictive value and clinical applicability, which might be useful for CAS screening in developing countries, including China.

This study has some inevitable limitations. First, the inclusion of the study population was regional, which may have affected the extrapolation of the prediction model. Second, the collection of clinical baseline data was not sufficiently comprehensive, and potential clinical predictors may have been overlooked. Third, the prediction models were constructed based on cross-sectional studies, and the stability of the models must be tested in clinical practice. In future studies, we will cooperate with multiple centers to continuously test and revise the prediction model during clinical practice and further improve its extrapolation of the prediction model.

## Conclusion

This study identified eight CAS-associated independent risk factors, including age, BMI, DBP, WBC, MPV, ALT, AST, and GGT, based on the Chinese population. Meanwhile, we developed risk models for identifying individuals at high risk of CAS, which is important for preventing and reducing adverse prognostic events.

## Data availability statement

The original contributions presented in this study are included in the article, further inquiries can be directed to the corresponding author.

## Ethics statement

The study was approved by the ethics committee of the Affiliated Hospital of Medical School, Ningbo University, Ningbo, China (KY20191114). The patients/participants provided their written informed consent to participate in this study.

## Author contributions

GH conceived and designed the research and drafted the manuscript. GH and QJ did the statistical analysis. GH and XT took part in the discussion. YM revised the manuscript. All authors read and approved the final manuscript.

## Footnotes

1
https://www.R-project.org
